# The Stability–Release Paradox in Long-Acting Peptide and Protein Delivery: A Review of Material–Cargo Compatibility and Therapeutic Integrity

**DOI:** 10.3390/pharmaceutics18101306

**Published:** 2026-10-15

**Authors:** Sohail Mumtaz, Amir Muhammad Afzal

**Affiliations:** 1 Department of Chemical and Biological Engineering, Gachon University, 1342 Seongnamdaero, Sujeong-gu, Seongnam-si 13120, Republic of Korea; 2 Department of Physics, University of Okara, Okara 56300, Pakistan

**Keywords:** long-acting injectables, peptide therapeutics, protein therapeutics, hydrogel depots, PLGA microspheres, therapeutic integrity, sustained release

## Abstract

Long-acting peptide and protein delivery requires more than prolonged release; the therapeutic must remain chemically, physically, structurally, and functionally competent throughout processing, depot residence, and release. This review examines the stability–release paradox, in which material features introduced to extend residence can also alter cargo integrity. Rather than cataloging platforms or restating the need for biologic stabilization, the literature is organized around a cargo-specific compatibility question: whether a retention mechanism, depot microenvironment, and intended residence time remain compatible with preservation of therapeutic attributes required for activity. Published experimental studies and recent reviews available through 10 August 2026 were critically examined, with emphasis on poly(lactic-co-glycolic acid) (PLGA) systems, hydrogels, affinity-based depots, self-assembling formulations, and translational studies linking release with stability or pharmacological performance. Across these platforms, integrity is influenced by hydration, microclimate pH, reactive degradation products, drug–material affinity, interfacial exposure, local concentration, molecular crowding, processing stress, and host–depot interactions. Experimental studies of degradable polyesters show that prolonged mass release can coexist with peptide acylation or other chemical changes, whereas hydrogel and affinity systems demonstrate that reversible retention can suppress premature escape but still requires cargo-specific verification of recovery and biological function. The review therefore frames long-acting formulation design around three linked considerations: the depot as a dynamic pharmaceutical microenvironment, therapeutically competent release as an endpoint beyond cumulative mass recovery, and residence-time–stability matching as a compatibility test between planned confinement and the cargo’s demonstrated stability window. This framework supports material selection and evaluation based on therapeutic integrity, stability-indicating release measurements, clinically relevant loading, and pharmacokinetic/pharmacodynamic (PK/PD) evidence appropriate to the therapeutic claim.

## 1. Introduction

Peptide and protein therapeutics are increasingly important in modern pharmacology. Their molecular recognition can access targets that are difficult to address with many conventional small molecules. The functional roles of peptides are also expanding beyond conventional receptor agonism and antagonism, with peptide ligands increasingly being explored as molecular-recognition elements in targeted protein-degradation strategies [1]. Clinical value, however, is often coupled to short systemic persistence, proteolytic susceptibility, limited permeability, or administration by injection. Molecular half-life extension can address some of these constraints, but it does not eliminate the need for sustained local or systemic exposure. Long-acting materials, therefore, remain attractive when dosing frequency, adherence, local retention, or exposure control are central product goals [2,3,4,5,6,7,8,9,10].

The materials literature has made substantial progress in controlling release. Biodegradable microspheres and implants use diffusion, polymer erosion, or combinations of both; hydrogels exploit mesh-controlled diffusion, reversible affinity, dynamic networks, or composite architectures; self-assembling therapeutics can create their own reservoirs. These platforms can convert a solution-phase drug into a depot that persists for days to months. Yet the dominant performance metric is still frequently a cumulative release curve. That convention is reasonable for a chemically robust cargo, but it becomes scientifically insufficient when the therapeutic itself is structurally labile. A depot can release the correct mass at the correct rate and still fail pharmaceutically if the released population contains chemically modified, aggregated, unfolded, or functionally compromised species.

Long-acting formulation science now has a substantial translational literature spanning PLGA depots, in situ systems, hydrogels, implants, release testing, and patient-centered product design [11,12,13,14,15,16,17,18,19]. These studies show that duration is governed by coupled material, process, analytical, and in vivo constraints. The challenge becomes sharper for peptides and proteins because processing and prolonged material residence can change cargo quality [11,14]. An integrity-centered framework is therefore needed to connect sustained release with pharmaceutical performance.

The general requirement that an extended-release formulation preserve drug activity is not unique to peptides and proteins and is not, by itself, the novelty addressed here. The more specific problem is that the same material features used to prolong residence can alter the pharmaceutical state of the retained therapeutic, creating a mechanistic coupling between release control and cargo integrity. This coupling is particularly consequential for peptide and protein therapeutics because mass recovery alone may not reveal covalent modification, self-association, aggregation, conformational perturbation, or loss of target-specific activity. Folded proteins add higher-order structural and interfacial sensitivities, whereas peptides present sequence- and chemistry-dependent liabilities that can include hydrolysis, oxidation, deamidation, epimerization, or polymer-associated reactions [2,20]. Consequently, successful transfer of a retention strategy from one therapeutic to another cannot be assumed solely from similar release kinetics.

Previous reviews have comprehensively described long-acting carrier technologies, peptide loading and release strategies, and approaches for mitigating protein and peptide instability [8,9,10,11,12,13,14,15,16,17,18,19,21,22]. The present review is therefore not intended as another inventory of delivery platforms or a restatement that biologics require stabilization. Instead, it reorganizes the experimental evidence around a formulation question that remains insufficiently resolved: which combinations of therapeutic liability, retention mechanism, depot microenvironment, and residence time remain compatible with release of a therapeutically competent molecular population? To make this distinction useful for formulation scientists, the analysis links experimentally observed material–cargo failure modes to carrier-selection and mitigation strategies, distinguishes mass release from progressively stronger evidence of chemical, structural, functional, and in vivo integrity, and evaluates whether the intended residence period is compatible with the experimentally demonstrated stability window of the therapeutic. We therefore define the stability–release paradox as an engineering tension. Stronger or longer confinement can improve release control, yet it can also increase interfacial contact, local concentration, residence time, or chemical exposure for a labile therapeutic. Prolonged retention is not intrinsically damaging. The framework instead asks whether a retention mechanism is compatible with the specific molecular liabilities of the cargo.

Throughout this Review, therapeutically competent release is used as a proposed design concept, not as a regulatory category. It denotes a released therapeutic that meets drug-appropriate criteria for chemical identity and purity. It also requires adequate physical or higher-order structural integrity and biological function. In vivo exposure and pharmacodynamic effect should be assessed when the intended claim requires them. Residence-time–stability matching is likewise proposed as a compatibility test. The intended residence or release interval should remain within the period over which relevant quality attributes are acceptably preserved under local depot conditions. If it does not, a protective strategy must change that stability window.

**Figure 1:**
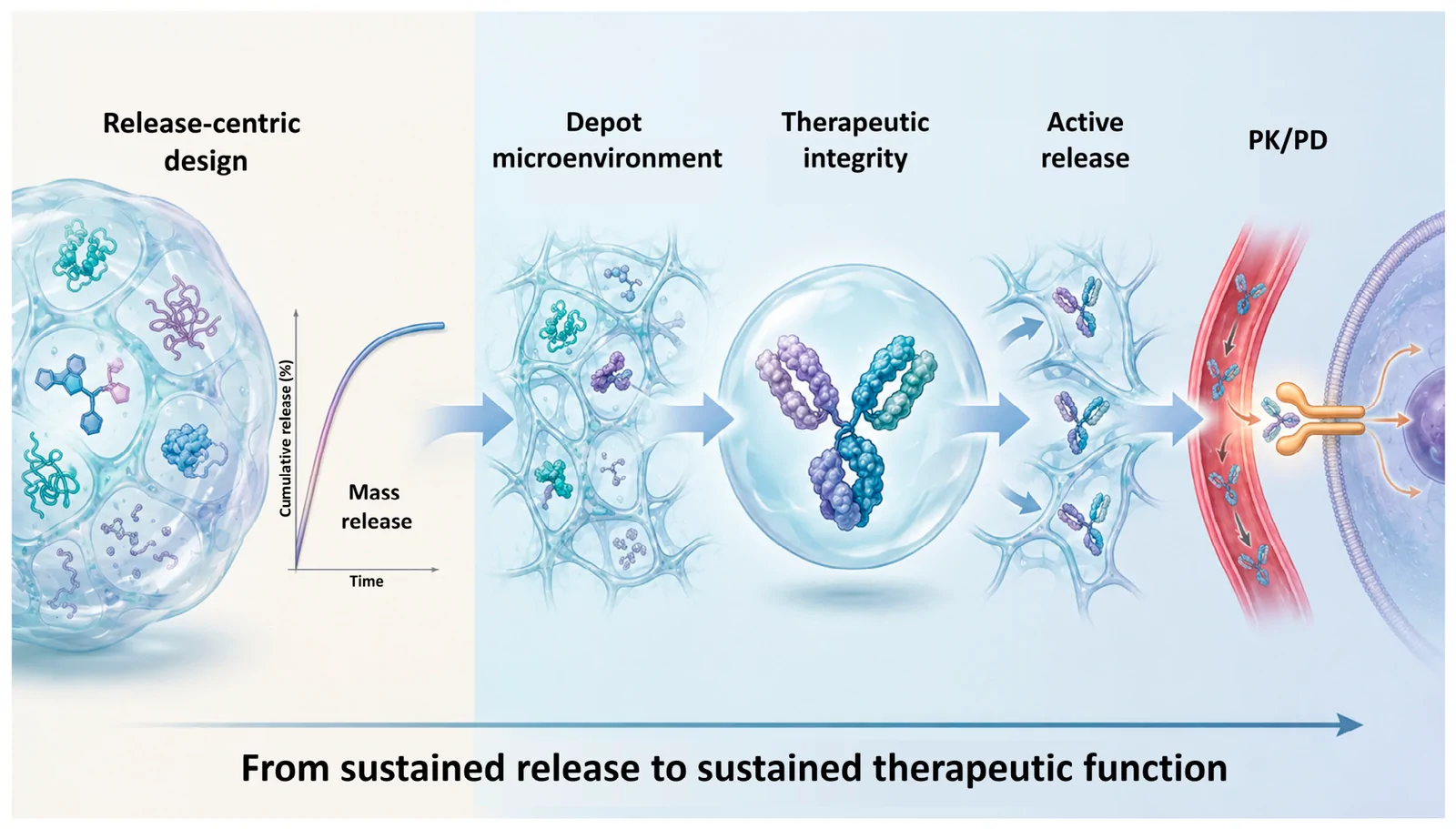
From sustained release to sustained therapeutic function. Conceptual comparison of release-centered depot design with an integrity-centered approach integrating the depot microenvironment, therapeutic integrity, release of active drug, and downstream PK/PD performance.

The scope is intentionally restricted to material-based long-acting delivery of peptides and proteins, including enzymes, growth factors, antibody fragments, and monoclonal antibodies. This boundary does not imply that retention–stability trade-offs are absent from small molecules, nucleic acids, cellular products, or other therapeutic modalities. Although some formulation and stability challenges overlap across therapeutic modalities, their dominant delivery barriers and performance endpoints are not equivalent. Nucleic-acid therapeutics require protection against nuclease-mediated degradation and, for many mRNA/siRNA systems, productive cellular uptake followed by endosomal escape into the cytosol [23,24]. Delivery systems for living-cell therapeutics must additionally preserve cell viability and functional phenotype while supporting survival, engraftment, and appropriate interactions with the host environment [25]. Oral controlled-release systems face route-specific gastrointestinal barriers, including variable pH, enzymatic degradation, mucus, and limited epithelial permeability [26]. These modality- and route-specific requirements demand different material-design criteria and analytical endpoints from those emphasized here for prolonged material residence and therapeutic integrity of peptide and protein depots. Route-specific delivery systems can likewise be governed by transport barriers that differ from prolonged depot residence; for example, engineered exosomal formulations have been developed to enhance transdermal delivery of immunomodulatory cargo for psoriasis therapy [27]. Rather, combining these mechanistically distinct cargo classes would obscure the cargo-specific relationship between material residence and pharmaceutical integrity that is the focus of this Review. Peptides and proteins provide a defined evidence domain in which chemical modification, higher-order structural perturbation, aggregation, material-interface effects, biological activity, and pharmacokinetics/pharmacodynamics (PK/PD) can be connected directly to depot design. Intracellular protein delivery introduces an additional requirement for productive cytosolic access that is distinct from prolonged extracellular depot residence; for example, a recent polyphenol-based nanoplatform protected a therapeutic enzyme from degradation while preserving its structure and enzymatic activity and enabling triggered cytosolic release [28]. Nucleic acids, cellular therapies, conventional oral controlled-release systems, and generic nanoparticle targeting are therefore outside the core analysis except where they provide directly relevant mechanistic context. For example, receptor-targeted liposomal systems can be engineered primarily to overcome anatomical transport barriers, as illustrated by cRGD-modified liposomes designed to enhance blood–tumor-barrier penetration in glioblastoma [29]; such targeting objectives are mechanistically distinct from maintaining peptide or protein integrity during prolonged depot residence. Figure 1 summarizes the resulting shift from controlling release duration alone to engineering sustained therapeutic function.

### 1.1. Literature Identification Approach

A focused critical literature assessment was undertaken to identify studies that directly interrogate the coupling between depot design, therapeutic integrity, and long-acting performance. Literature was identified through searches of PubMed/MEDLINE and Google Scholar using terms related to peptide and protein depots, PLGA and hydrogel systems, affinity-mediated retention, material–drug interactions, stability and degradation, release kinetics, and pharmacokinetics/pharmacodynamics (PK/PD). Because the review was designed as a focused, hypothesis-driven synthesis rather than a formal systematic review, searches were performed iteratively using combinations of concept-specific terms rather than a single prespecified search string. Boolean operators AND and OR were used to connect the principal search domains. Representative combinations included (“peptide” OR “protein”) AND (“long-acting” OR “depot” OR “sustained release”) AND (“PLGA” OR “hydrogel” OR “affinity-mediated” OR “self-assembling”); (“peptide” OR “protein”) AND (“PLGA” OR “hydrogel”) AND (“stability” OR “degradation” OR “aggregation” OR “oxidation” OR “deamidation”) AND (“release” OR “release kinetics”); and (“peptide” OR “protein”) AND (“material–drug interaction” OR “polymer–drug interaction” OR “adsorption” OR “acylation”) AND (“bioactivity” OR “potency” OR “therapeutic integrity”). Additional searches combined long-acting peptide or protein delivery with pharmacokinetic, pharmacodynamic, and translational terms. Search syntax was adapted as necessary to the individual database or search platform.

Primary experimental studies were prioritized when they provided mechanistic or translational evidence linking material properties, drug–material interactions, depot microenvironment, therapeutic stability or degradation, release behavior, biological activity, or in vivo pharmacological performance. Recent review articles and regulatory or methodological guidance were used primarily to establish context, identify relevant primary studies, and define unresolved translational gaps. No lower publication-date cutoff was imposed because earlier foundational studies remain important for mechanisms such as PLGA microclimate acidification, peptide acylation, and protein stabilization; the evidence base was updated through 10 August 2026. Detailed critical synthesis was restricted to publications for which an English-language full text was available. Studies centered on therapeutic modalities outside the defined peptide/protein scope, including nucleic-acid and cellular therapies, conventional oral controlled-release systems, and generic nanoparticle-targeting approaches, were not included in the core evidence synthesis unless they provided directly relevant mechanistic or methodological context. Studies were also deprioritized when they reported sustained mass release without information relevant to material–cargo interactions, therapeutic stability, molecular integrity, biological function, or translational performance. Accordingly, the objective was a transparent, concept-driven critical synthesis rather than exhaustive evidence enumeration or a formal PRISMA-style systematic review.

### 1.2. Methods: Use of Generative Artificial Intelligence

**Figure 2:**
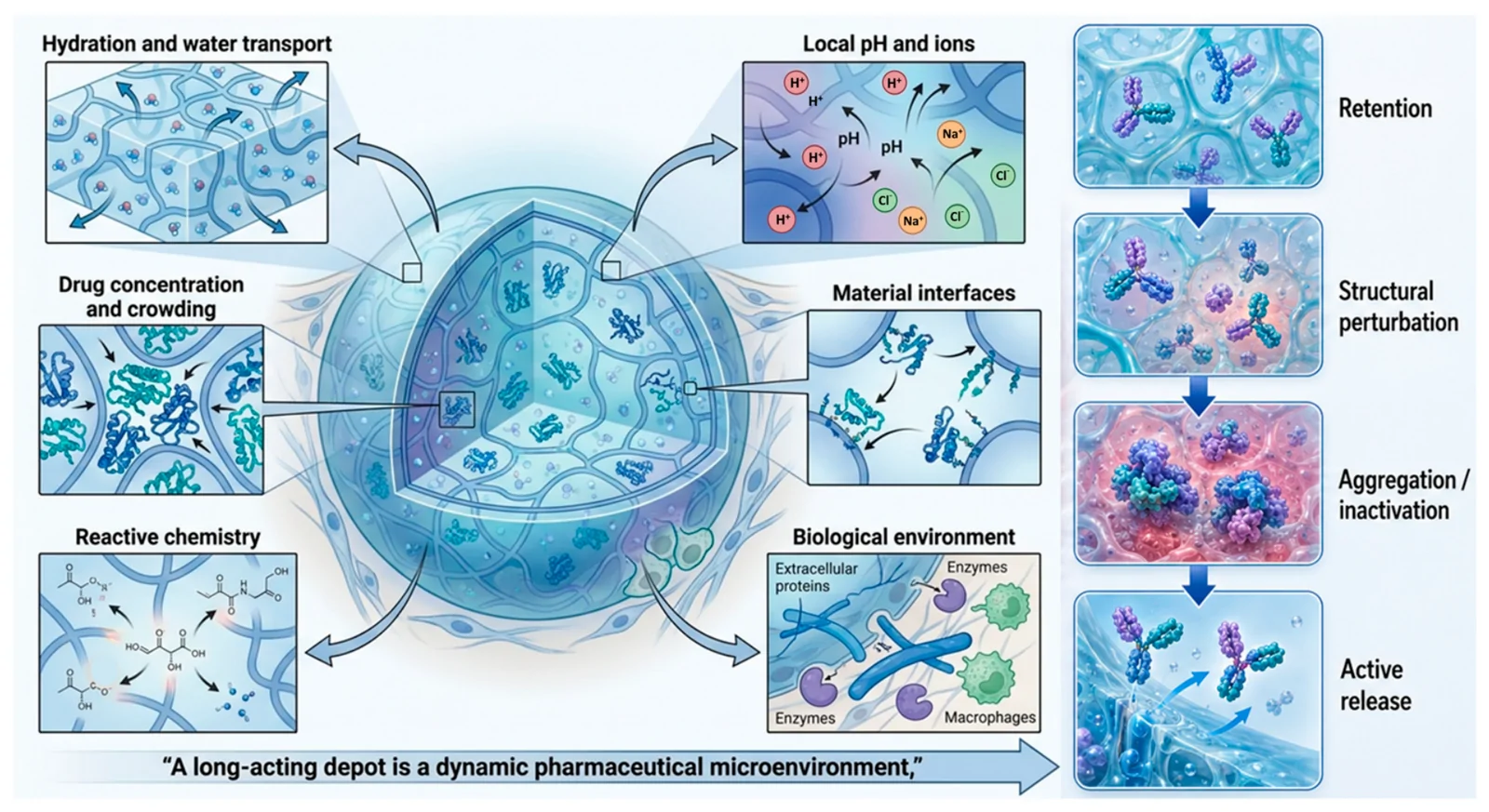
Dynamic pharmaceutical microenvironment within a long-acting peptide and protein depot. A material depot creates an evolving local environment influenced by hydration and water transport, local pH and ionic conditions, drug concentration and molecular crowding, material interfaces, reactive chemistry, and interactions with the surrounding biological environment. The balance among these factors determines whether the retained therapeutic remains stable or undergoes structural perturbation, aggregation, chemical modification, or functional loss before release.

**Figure 3:**
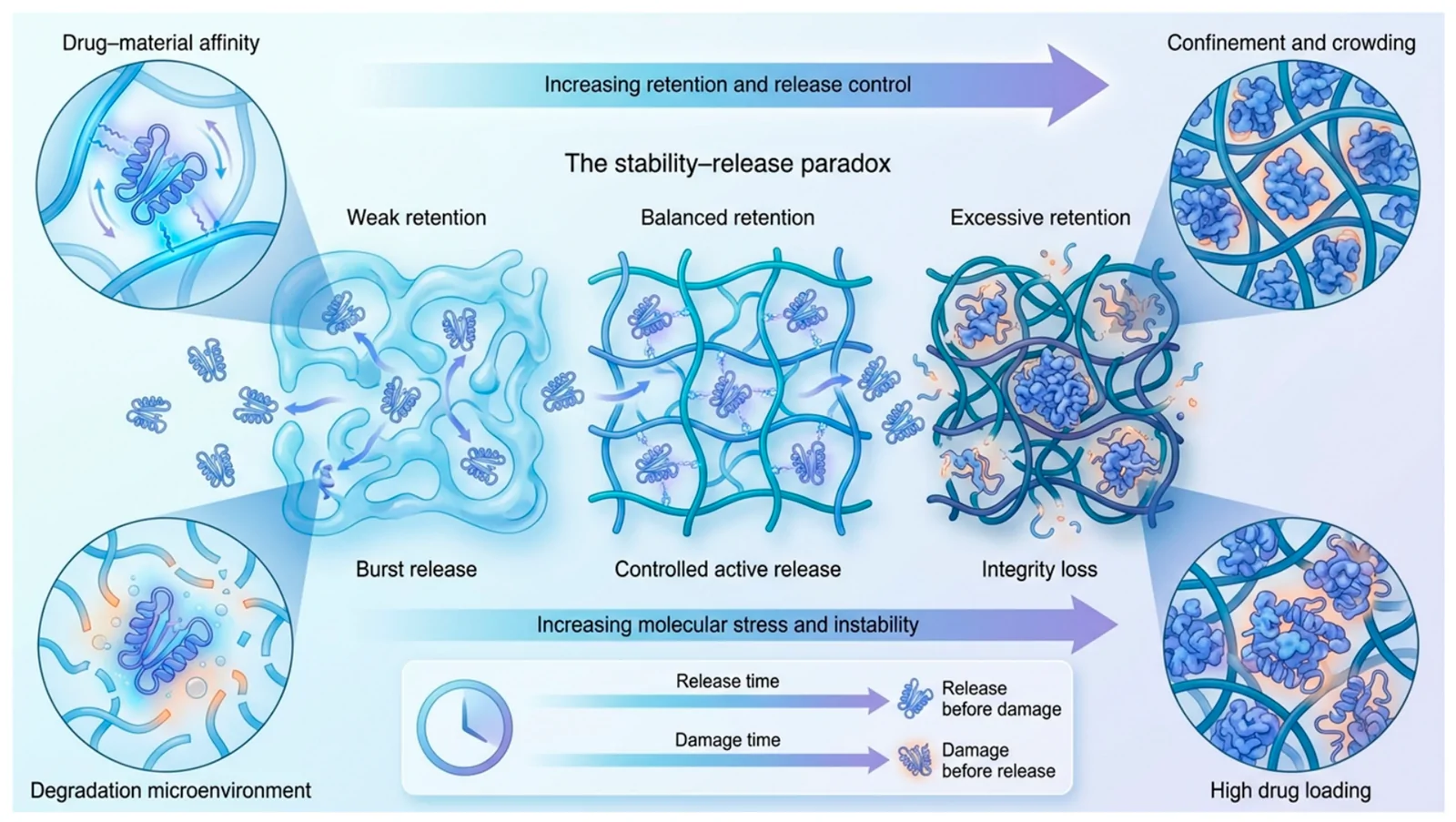
The stability–release paradox in long-acting peptide and protein delivery. Weak retention can result in rapid escape or burst release, whereas excessive confinement may increase drug–material interactions, molecular crowding, local concentration, and exposure to degradation-associated microenvironments. Optimal depot performance therefore requires a compatibility window in which retention is sufficient to control release but the intended residence period remains within the therapeutic stability window.

**Figure 4:**
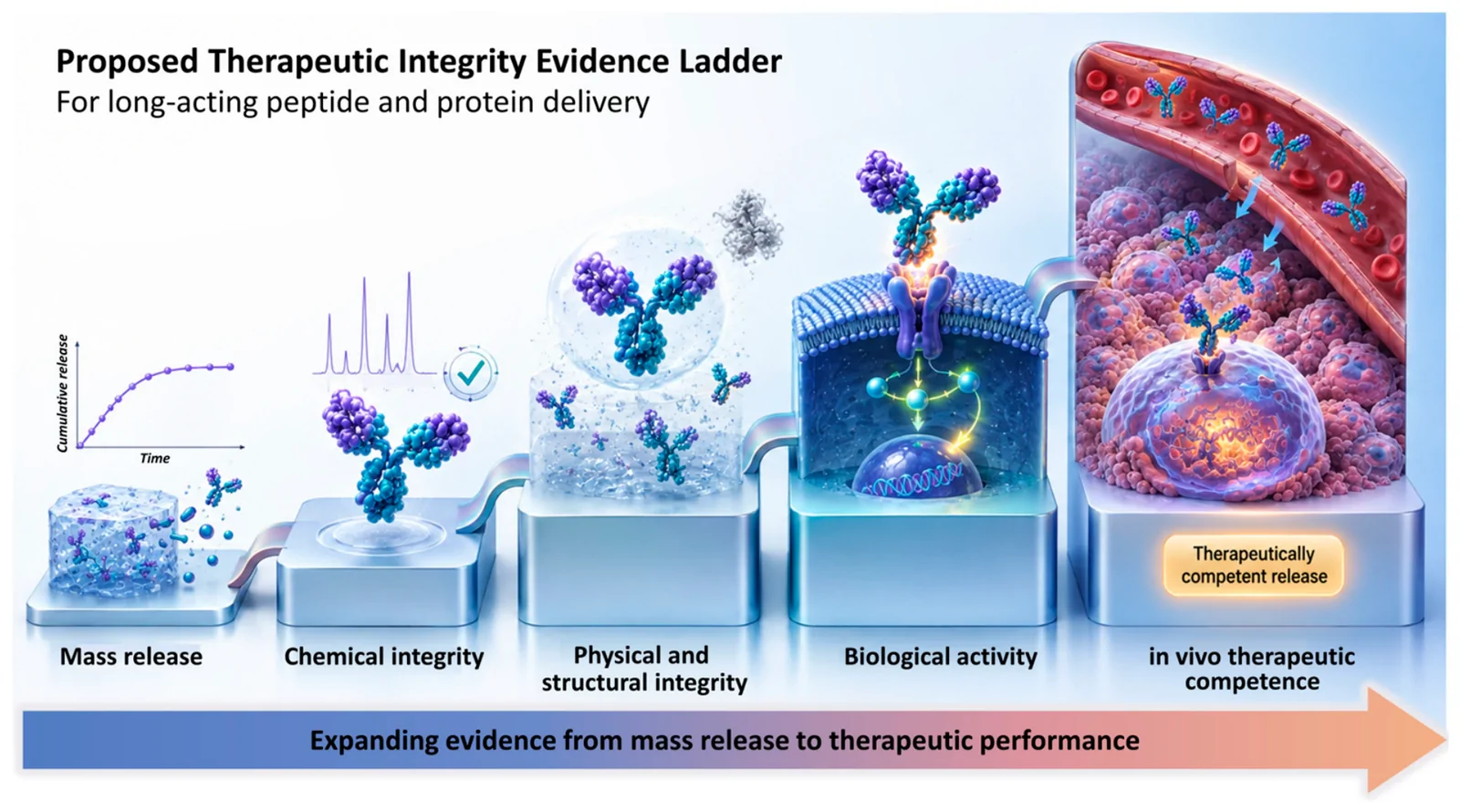
Proposed Therapeutic Integrity Evidence Ladder for long-acting peptide and protein delivery. The framework organizes evidence from cumulative mass release through chemical integrity, physical and structural preservation, biological activity, and in vivo therapeutic competence. The levels represent distinct evidence domains rather than a validated universal sequence or mandatory analytical checklist, and the assays required at each stage remain cargo- and claim-dependent. The framework is intended to distinguish what has been experimentally demonstrated from what remains unmeasured when evaluating claims of sustained therapeutic delivery.

**Figure 5:**
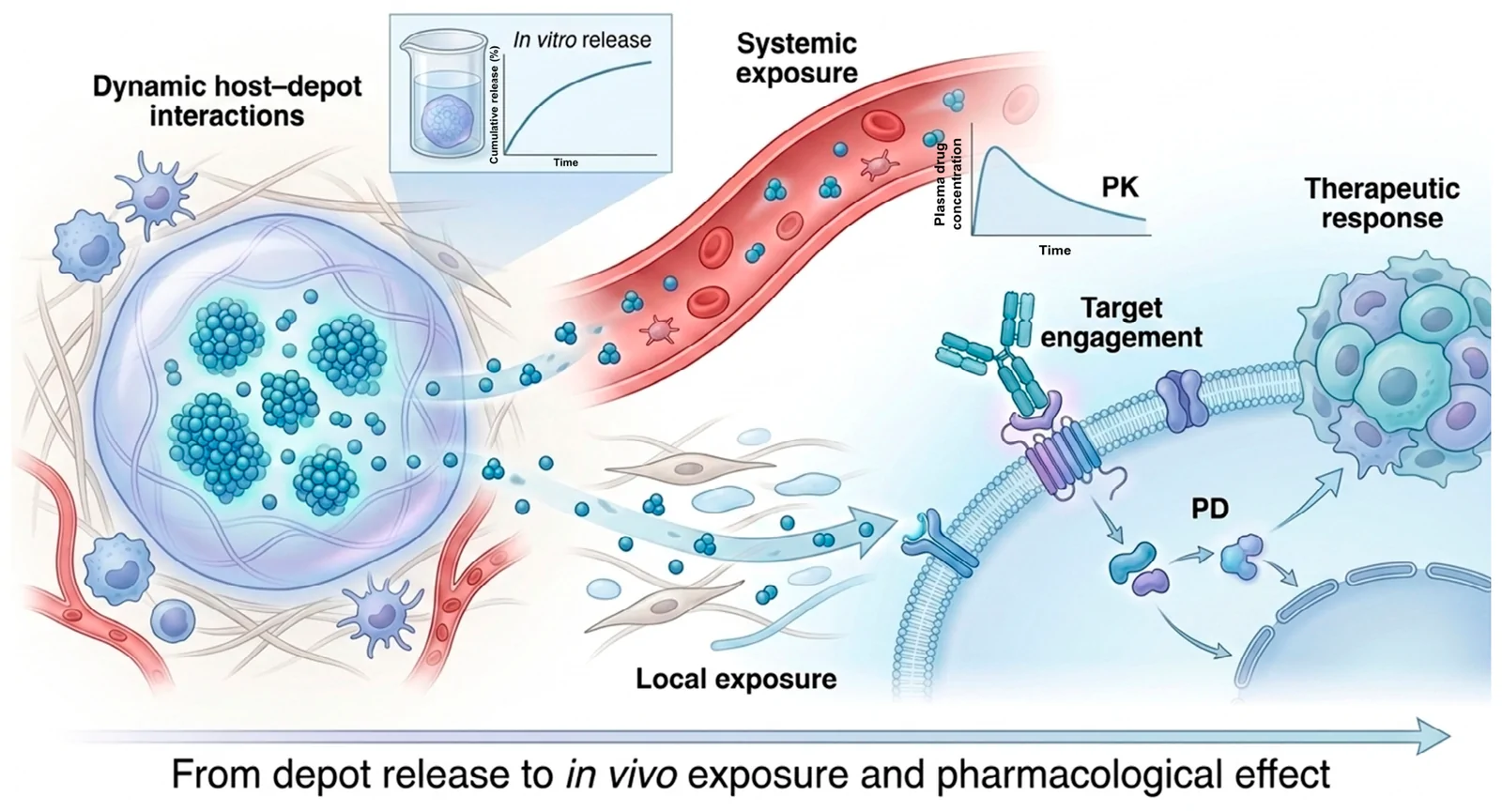
From depot release to in vivo exposure and pharmacological effect. Following administration, depot behavior is influenced by the surrounding tissue environment and determines the amount and rate of therapeutically competent drug reaching local and/or systemic compartments. These exposure pathways shape pharmacokinetics (PK), target engagement, downstream pharmacodynamic (PD) responses, and therapeutic outcome. The scheme therefore connects material-controlled release with the biological processes that ultimately determine in vivo performance.

**Figure 6:**
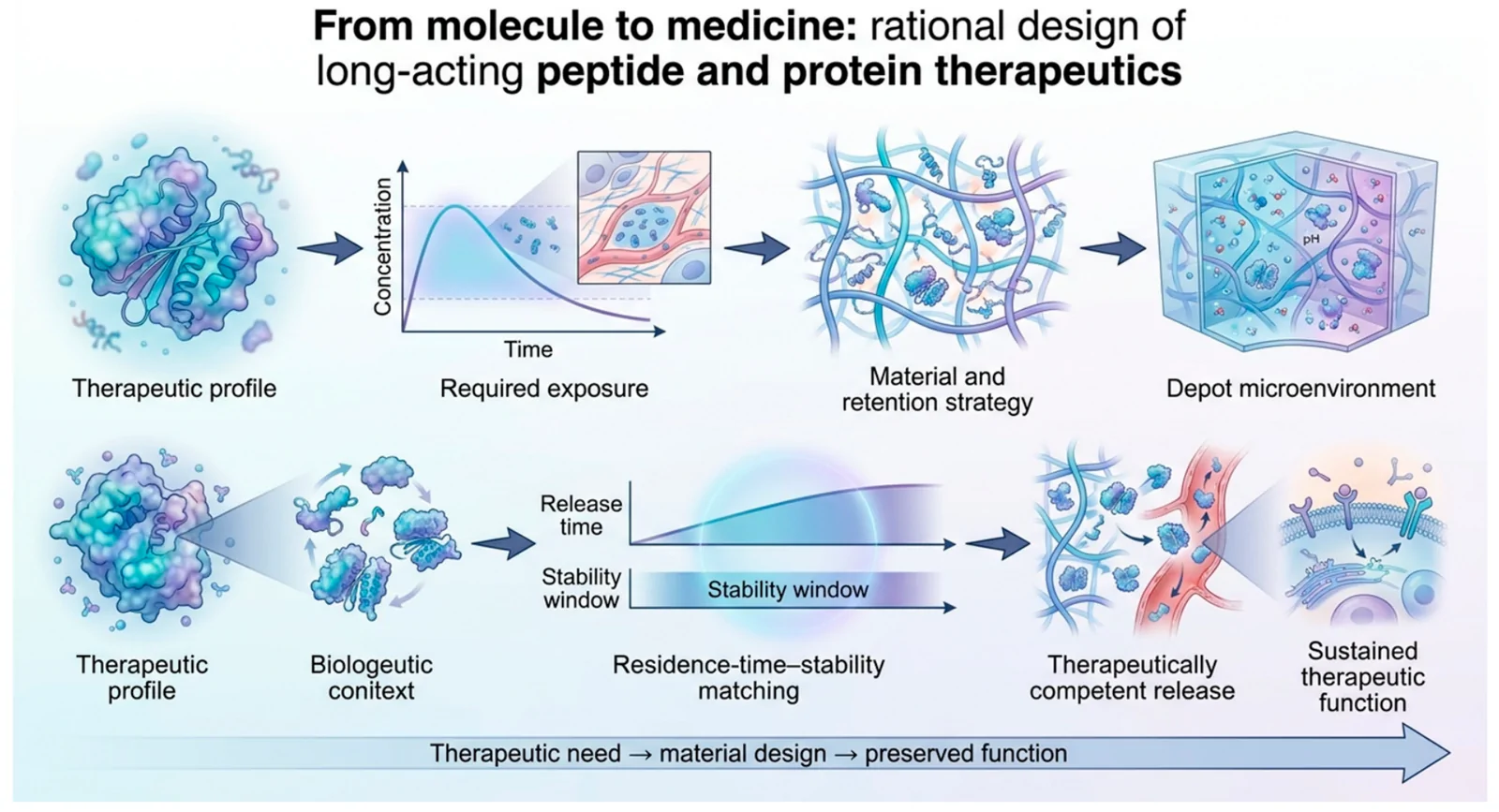
Target-product-profile-driven framework for rational design of long-acting peptide and protein therapeutics. Rational development begins with the therapeutic properties, intended dose, dosing interval, and required exposure profile. These requirements guide material and retention-strategy selection, engineering of the depot microenvironment, and residence-time–stability matching. Successful alignment of these elements should enable therapeutically competent release, appropriate target exposure, and sustained therapeutic function while remaining compatible with translational and product-development constraints.

During the preparation of this work, all literature searches, critical evaluation of studies, data synthesis, interpretation of findings, and initial drafting of the manuscript were performed manually by the authors. The fundamental concepts and scientific conclusions presented in this review were derived solely from the authors’ independent analysis of the published literature. ChatGPT (OpenAI, version GPT-5.6 Sol, San Francisco, CA, USA) was subsequently used solely to improve language quality, simplify and reconstruct sentences, and enhance the clarity and readability of the manuscript, including assisting in the presentation of complex mechanistic concepts in a more accessible manner for readers. FigureLabs was used selectively through an author-held subscription account to assist with the initial graphical drafting of Figure 1 and Figures 2–6 based on the authors’ original concepts and scientific interpretations. Microsoft PowerPoint was subsequently used for manual editing and final adjustments where necessary. All figures were critically reviewed and verified by the authors to ensure scientific accuracy, clarity, and consistency with the manuscript. After using these tools, the authors reviewed and edited the content as needed and took full responsibility for the publication’s content.

## 2. Therapeutic Integrity as a Design Constraint

### 2.1. Molecular Complexity Expands the Number of Critical Attributes

Cargo class alone does not predict compatibility with a long-acting material. A particularly instructive example is octreotide. In PLGA microspheres, approximately 85% of the peptide loading was eventually released over 95 days, which could appear satisfactory if only cumulative release were considered. However, only 25% of the released peptide remained unmodified, whereas approximately 75% consisted of acylated products. When the carrier chemistry was changed to the more hydrophilic poly(D,L-lactide-co-hydroxymethyl glycolide) (PLHMGA), sustained release was retained, but the fraction of native octreotide increased substantially; depending on copolymer composition, more than 65–70% of the released peptide remained native [30]. In contrast, a substantially larger Fab fragment formulated as spray-dried solid protein in an injectable PLGA–triacetin depot showed sustained release for nearly 80 days while retaining more than 80% antigen-binding capacity after several weeks [31]. These contrasting cases demonstrate why molecular size alone is a poor predictor of depot compatibility: a short peptide can undergo extensive carrier-mediated covalent modification, whereas a folded antibody fragment can remain functionally competent in a suitably engineered polyester environment.

The relevant integrity attributes should therefore be defined from the known liabilities of the individual therapeutic and the chemistry of its retention environment. For a nucleophilic peptide residing in a degrading polyester, stability-indicating chromatography and mass spectrometry may be more informative than total peptide recovery because polymer-associated acylation can dominate the failure mode [30]. For folded proteins and antibody fragments, monomer state, higher-order structure, and target-specific binding or potency become increasingly important [31,32,33,34]. High-concentration monoclonal-antibody systems add viscosity, self-association, and interfacial behavior as formulation constraints [35,36,37]. Molecular complexity therefore expands the number of potentially critical attributes but does not establish a universal ranking of fragility; the relevant failure mode remains molecule-, formulation-, concentration-, and pathway-specific.

This distinction matters because a single analytical readout can create false confidence. Total protein recovered from a release medium can remain high while monomer content falls. An immunoassay can retain signal despite chemical modifications outside the recognized epitope. Conversely, a modest conformational change detected spectroscopically may not necessarily translate into potency loss. Therapeutic integrity must therefore be defined prospectively from the molecular features that determine the intended function rather than retrospectively from whichever assay is easiest to perform.

### 2.2. A Hierarchy of Integrity

For long-acting depots, five nested levels are useful. Chemical integrity asks whether covalent identity and purity are maintained, including oxidation, deamidation, hydrolysis, clipping, acylation, and other product-related variants. Physical and colloidal integrity addresses soluble and insoluble aggregates, precipitation, particles, phase separation, and self-association. Structural integrity concerns higher-order conformation when that conformation is required for function. Functional integrity asks whether the released material still binds its target, catalyzes its reaction, or produces the expected cell-based response. Therapeutic integrity is the integrated pharmaceutical state required to support the intended exposure, potency, and safety profile.

Aggregation illustrates why these levels cannot be collapsed. Protein aggregation can arise through partial unfolding, protein–protein association, interfacial adsorption, mechanical perturbation, and formulation conditions [33,38,39,40,41,42,43,44,45,46,47,48,49,50,51,52,53,54]. Aggregate and particle formation can reduce usable drug and increase product-quality concerns. Some aggregate or particle states can also influence immunogenicity risk, but the relationship is context-dependent [55,56,57,58]. High-concentration formulations make this problem more demanding because molecular crowding, viscosity, and self-association are coupled [35,36,37,59,60,61,62,63,64,65,66,67].

The pharmaceutical formulation literature provides a second layer of evidence. Protein adsorption at solid–liquid and air–liquid interfaces can alter recovery, conformation, and particle formation [39,51,52,53,54,68]. Surfactant degradation, residual impurities, and container or process interfaces can further modify stability risk [40,41,42,43,44,45]. Protein instability can also persist in concentrated or solid states [46,69,70]. These mechanisms are not depot-specific, but they define hazards that a long-residence material must either avoid or explicitly test.

### 2.3. Therapeutically Competent Release

The proposed concept of therapeutically competent release is intended to correct a recurring mismatch between what is measured and what is claimed. Level 1 evidence is mass release: how much nominal drug leaves the depot. Level 2 is a chemically intact release. Level 3 establishes physical and structural integrity with appropriate orthogonal methods. Level 4 demonstrates drug-specific biological function. Level 5 connects the released species to in vivo pharmacology. Not every study needs every level, but the strength of the claim should not exceed the level of evidence. A paper claiming tunable diffusion may reasonably stop at release kinetics; a paper claiming successful sustained delivery of an antibody for therapy should show more than total protein recovery.

The consequence for materials design is profound. The ‘best’ matrix is not the material that minimizes diffusion most aggressively. It is the material that delivers the required exposure while preserving the relevant quality attributes of the chosen cargo. This cargo-specific framing also explains why universal recipes are unlikely. A hydrophobic interaction may efficiently retain one peptide but perturb another. A mildly acidic microenvironment may be tolerated by one protein yet accelerate modification of another. A mesh size optimized for an antibody fragment may release a smaller peptide too rapidly. Table 1 compares the major cargo-specific integrity liabilities that should be treated as design inputs rather than post hoc analytical observations.

Representative experimental cases linking cargo identity, carrier architecture, integrity outcome, and formulation consequence are compared in Table 2.

## 3. The Depot as a Dynamic Pharmaceutical Microenvironment

A long-acting depot is often drawn as a static sphere or gel from which the drug diffuses into an infinite sink. In vivo, it is neither static nor compositionally uniform. Water penetrates, and polymers hydrate or erode. Ions and proteins partition into the material. Local pH can diverge from that of the surrounding tissue. Therapeutic molecules may become concentrated in specific domains, while host cells can infiltrate or remodel the depot. Together, these processes define a local pharmaceutical microenvironment. That microenvironment may be more relevant to cargo stability than the nominal formulation composition.

### 3.1. Water Ingress, Degradation, and Microclimate pH

PLGA provides one of the clearest demonstrations that nominal material composition does not define the environment actually experienced by an entrapped therapeutic. Degradation generates acidic oligomers and monomers that can accumulate within poorly exchanging aqueous pores, and confocal studies have demonstrated spatially heterogeneous microclimate-pH distributions [86,87,88,89]. Importantly, Hong et al. extended this analysis in vivo using exenatide-loaded 50:50 PLGA microspheres implanted subcutaneously in rats for up to 6 weeks [82]. The in vivo microclimate-pH evolution resembled a low-microsphere-concentration in vitro condition but differed markedly from the conventional concentrated sample-and-separate release condition. Mass-spectrometric analysis of exenatide remaining within the particles simultaneously showed progressive degradation dominated by pH-dependent acylation [82]. This experiment directly connects an evolving carrier microenvironment with chemical transformation of a specific retained therapeutic and also demonstrates that the release-test geometry itself can alter the apparent microenvironment. External physiological pH therefore cannot be assumed to represent the conditions experienced by cargo inside a degrading depot.

### 3.2. Reactive Polymer–Drug Chemistry

Peptide susceptibility to reactive polyester degradation products is strongly dependent on molecular structure. Na et al. compared salmon calcitonin (sCT), human parathyroid hormone 1–34 (hPTH1–34), and leuprolide incorporated into degrading PLGA microspheres [72]. Capillary electrophoresis and MALDI-TOF mass spectrometry identified acylation products of sCT and hPTH1–34 as peptide adducts containing glycolic-acid units derived from PLGA degradation. In contrast, no corresponding acylation product was detected for leuprolide after 28 days of incubation [72]. This difference was associated with peptide primary structure: the susceptibility of sCT and hPTH1–34 was linked principally to accessible primary amino groups, whereas leuprolide lacks a free primary amine despite containing hydroxyl-bearing residues. The comparison demonstrates that chemically similar depot conditions can produce markedly different stability outcomes among peptides. Thus, the risk of polymer-mediated covalent modification should be assessed from cargo-specific reactive groups together with the chemistry of the degrading carrier, rather than inferred from release duration or polymer class alone.

### 3.3. Interfaces, Crowding, and Local Concentration

Hydrogels avoid some stresses associated with hydrophobic polyesters and organic-solvent microencapsulation, but an aqueous matrix is not automatically a benign protein formulation. Polymer–water interfaces, hydrophobic substituents, charged domains, residual crosslinking chemistry, and high local protein concentrations can still alter behavior. Protein adsorption at interfaces can generate aggregates or particles [35,39,51,52,53,54,79]. Surfactant chemistry, impurities, and packaging-related interfaces can further modify interfacial risk [40,41,42,43,44,45,52]. These observations are mechanistically supportive rather than proof that every hydrogel interface destabilizes an antibody. They justify testing the actual material–protein interface instead of assuming compatibility from bulk composition.

Local crowding can also be created deliberately. A high dose per injection volume is desirable for many subcutaneous antibodies. Concentrated protein states, however, introduce viscosity, self-association, solubility, and manufacturing constraints [35,36,37,59,60,61,62,63,64,65,66,67]. A depot can intensify this problem by creating microscale concentration gradients as water redistributes or drug partitions into domains. The stability-relevant concentration may therefore be the local concentration inside the depot, not the nominal concentration used during preparation.

### 3.4. Host Biology Is Part of the Microenvironment

The most important conceptual update is that the host is not an external boundary condition. Jons and colleagues showed that cellular infiltration into a polymer–nanoparticle hydrogel could actively increase antibody transport and that co-formulating an anti-inflammatory agent reduced infiltration and prolonged pharmacokinetic exposure [81]. This is direct evidence that host–material interactions can become part of the release mechanism. In other systems, foreign-body reaction, extracellular enzymes, fluid exchange, fibrosis, and mechanical motion may similarly change the depot over time. Host effects, therefore, need to be treated as formulation variables when they materially alter release or stability, not merely as biocompatibility endpoints measured after formulation performance has been optimized.

### 3.5. Material Classes as Microenvironment Archetypes

Material classes are most useful when compared by the microenvironments they create. PLGA microspheres can sustain release for long periods, but labile cargo may encounter acidic, reactive, and evolving pores [30,31,34,71,72,73,74,75,76,77,82,86,87,88,89,90]. Hydrogels provide water-rich environments and gentle administration, yet hydrophilic proteins often need affinity or hierarchical structure to avoid rapid escape [22,51,52,53,91,92,93,94,95,96,97]. Affinity hydrogels use reversible binding to slow release without relying only on mesh restriction [84,98]. Self-assembling peptide or drug depots can reduce carrier burden but require control of burst, assembly state, and recovery [99]. Composite systems can separate cargo stabilization from bulk transport control, as illustrated by high-concentration solid antibody placed in alginate particles and then embedded in a thermogelling matrix [80]. Emerging inorganic carriers provide an additional materials archetype: layered double hydroxides can exploit electrostatic interactions, ion exchange, and pH-responsive behavior for controlled incorporation and delivery of bioactive macromolecules, including peptides and growth-factor-associated regenerative systems [100]. Figure 2 reframes the long-acting depot as a dynamic pharmaceutical microenvironment rather than an inert reservoir. Table 3 translates common material classes into microenvironmental advantages and liabilities rather than cataloging them by chemistry alone.

**Table 3:** Material microenvironment archetypes for long-acting peptide and protein delivery.

Archetype	Dominant Retention/Release Logic	Potential Advantage for Labile Cargo	Integrity Risk That Must Be Tested	Refs.
PLGA microspheres/implants	Diffusion + water ingress + polymer degradation/erosion	Long duration; established parenteral precedent	Acidic microclimate; peptide acylation; solvent/process stress; multiphasic release	[30,71,72,73,82,86,87,88,89,101]
Thermoresponsive/shear-thinning hydrogels	Mesh diffusion; physical association; in situ gelation	Aqueous processing; injectable; soft tissue compatibility	Fast escape of hydrophilic proteins; interface/crosslinker effects; dilution sensitivity	[22,92,102]
Affinity hydrogels	Reversible ligand–cargo binding + diffusion	Can slow release without extreme mesh restriction	Binding too weak → burst; too strong → incomplete recovery; cargo-specific perturbation must be assessed	[93,94,95,96]
Peptide/self-assembling depots	Therapeutic or carrier self-assembly controls dissolution/disassembly	High drug fraction; reduced carrier burden	Assembly heterogeneity; fibrillation/precipitation; burst upon disassembly	[78,99]
Hierarchical/composite depots	Separate cargo state from outer transport-controlling matrix	Allows independent control of stability, loading, injectability, and release	Greater manufacturing complexity; interfacial compatibility between subcomponents	[80,81,85]

The risk column identifies properties that require testing; it should not be read as evidence that every formulation in a class exhibits the listed failure mode.

## 4. The Mechanistic Core: The Stability–Release Paradox

### 4.1. Diffusion Control Versus Premature Escape

Highly hydrophilic peptides and proteins can escape rapidly from hydrated injectable matrices, limiting release duration even when the matrix itself provides a relatively mild aqueous environment [20,83]. Zhang et al. demonstrated this directly by comparing proteins incorporated into a PLGA–PEG–PLGA thermogel with the same proteins first encapsulated in dextran microparticles. Direct incorporation produced >25% initial release for G-CSF, GM-CSF, and BSA, whereas microparticle pre-encapsulation reduced release to ≤5% within 30 min [83]. For FVIII, first-day release decreased from >20% to <1%, while the released proteins retained ≥90% of their native biological activity [83]. These results show that when a hydrated matrix is intrinsically too permeable for a hydrophilic cargo, introducing an independently tunable transport barrier can suppress burst release without relying solely on greater network restriction. The design objective should therefore be sufficient retention with preserved recovery and activity, rather than minimum diffusivity alone [20,83].

### 4.2. Reversible Affinity: Retention Versus Recovery

Reversible affinity can supplement mesh-controlled diffusion and extend protein residence without covalently immobilizing the therapeutic. Isaacs-Bernal et al. incorporated an Fc-binding peptide ligand with an affinity of approximately 10^−8^ M into a hyaluronan hydrogel and demonstrated reduced burst release and effective diffusivity of Fc-Noggin and anti-sFRP2 relative to the corresponding non-affinity network [84]. Both therapeutics retained drug-specific biological activity after release, and a single affinity-hydrogel administration produced an in vivo retinal stem-cell response comparable to three repeated bolus administrations [84]. This study also illustrates why slower release alone should not be interpreted as successful retention: release kinetics should be considered together with cumulative recovery and post-release biological function. The practical objective is therefore reversible, function-preserving affinity rather than maximum binding strength [84].

### 4.3. Confinement and Loading Versus Crowding and Self-Association

High therapeutic loading can create a stability problem when increased protein concentration strengthens protein–protein or interfacial interactions. In a high-concentration monoclonal-antibody formulation, Escobar et al. showed concentration-dependent surface behavior and interface-induced particle formation, including reduced effectiveness of surfactant-mediated protection at the highest antibody concentration examined [35]. Long-acting formulations must therefore consider not only the nominal dose but also the physical state and local concentration of the therapeutic within the depot.

Zheng and Doyle addressed this challenge using IgG-loaded alginate microparticles containing approximately 213 mg/mL antibody, which enabled a final composite formulation concentration of 150 mg/mL [80]. Unmodified alginate microparticles released essentially all IgG within 1 h, whereas increasing hydrophobic modification reduced 1 h release to 44.7% and 17.2%; incorporation into the composite thermogel further prolonged release while maintaining antibody monomer content [80]. This architecture illustrates that high loading, protein physical state, and transport control can be engineered in separate formulation compartments rather than forcing a single homogeneous matrix to satisfy all requirements. For high-dose biologics, loading, aggregation or self-association, injectability, and release should therefore be evaluated as coupled formulation variables [35,80].

### 4.4. Degradation-Controlled Release Versus Degradation-Induced Damage

The stability–release paradox is most evident when degradation of the carrier is itself responsible for prolonged release, but the outcome cannot be predicted from the polymer name alone. Octreotide in conventional PLGA provides a failure case: prolonged release was obtained, yet most of the recovered peptide was chemically acylated [30]. In contrast, Johnson et al. stabilized recombinant human growth hormone as a zinc-containing solid and encapsulated it in PLGA using a cryogenic, non-aqueous process. Extracted rhGH remained analytically and biologically comparable to the pre-encapsulation protein, while subcutaneous microspheres maintained elevated serum rhGH for up to one month, more than 20-fold longer than the same amount administered as solution [34]. A third outcome was observed for spray-dried Fab1 in an injectable PLGA–triacetin depot, which released the antibody fragment for nearly 80 days while retaining more than 80% antigen-binding capacity after several weeks [31]. These cases show that PLGA should neither be rejected nor selected as a class. For nucleophilic peptides, controlling reactive polyester chemistry and the evolving microenvironment is critical; for folded proteins, solid-state stabilization and a protein-compatible manufacturing process may be equally important. Carrier selection should therefore be based on the experimentally demonstrated interaction between the specific cargo, processing route, polymer chemistry, and intended residence time rather than on generic assumptions about a material class.

### 4.5. Hydrophobic Retention and Interfacial Exposure

Hydrophobic interactions are often used to slow peptide or protein mobility. They can be introduced intentionally through functional groups or arise from partitioning into polymer domains. Protein formulation studies show that adsorption to hydrophobic or solid–liquid interfaces can promote conformational rearrangement and particle formation [33,35,38,47,48,49,50,51,52,53,79,86]. This does not mean that all hydrophobic depots are damaging. It does require a stricter experimental standard. When release control depends on adsorption or hydrophobic association, the recovered cargo should be tested for monomeric state and function. Material optimization should include interfacial stability, not release kinetics alone.

### 4.6. Processing Compatibility Versus Final Depot Performance

A depot begins exerting stress before it is injected. Double-emulsion microencapsulation can expose proteins to solvent interfaces, emulsification, osmotic shifts, and drying [11,14,20,31,34,76,77]. In situ-forming implants may expose cargo to organic solvent and phase inversion [14,17]. Reactive hydrogel crosslinking can create transient chemical environments that are absent from the final network. The material may look benign at equilibrium, even after manufacturing has modified part of the drug. Processing therefore belongs inside the stability–release problem, not outside it [40,41,42,43,44,45,51,52,70].

### 4.7. Residence-Time–Stability Matching

The preceding trade-offs can be unified by comparing intended residence with cargo stability under the relevant depot conditions. Consider a peptide designed to remain in a matrix for twelve weeks. If unacceptable covalent modification appears after three weeks at the internal pH and water activity of that depot, the formulation is mismatched. A protective strategy must extend the stability window before the long residence time can be considered useful.

This idea motivates residence-time–stability matching. The concept is not a universal kinetic constant and does not imply a single damage time. Different quality attributes can fail on different timescales. The practical question is simpler: does the therapeutic remain within its predefined integrity limits for the period required by the release design? Formulation teams should therefore measure time-dependent integrity while the drug remains inside the material. Those measurements should approximate the evolving in vivo microenvironment where possible. Figure 3 integrates the paradox into a compatibility window in which release control and therapeutic integrity are optimized simultaneously.

## 5. Engineering the Stability–Release Compatibility Window

### 5.1. Start with Drug-Friendly Loading and Fabrication

Protein stability can be compromised during depot preparation before the final release phase begins, particularly through exposure to organic solvents, interfaces, emulsification, drying, or unfavorable local environments [20]. Importantly, apparent mechanical damage should not automatically be attributed to shear itself. Thite et al. experimentally separated hydrodynamic shear from antibody adsorption at stainless-steel/water interfaces and showed that subvisible-particle formation could be suppressed by modifying the surface to reduce protein adsorption despite comparable high-shear conditions [79]. Their findings identify interfacial adsorption, rather than bulk shear alone, as a major contributor under the conditions studied [79].

Processing strategy can also determine whether a degradable polyester is compatible with a labile protein. Johnson et al. stabilized recombinant human growth hormone as a zinc-containing solid and encapsulated it in PLGA using a cryogenic, non-aqueous process [34]. The recovered rhGH remained comparable with the pre-encapsulation material by chromatographic, electrophoretic, peptide-mapping, and biological analyses, while a single microsphere administration maintained elevated serum rhGH for up to one month [34]. Formulation development should therefore identify and minimize the dominant cargo-specific processing stress before relying on downstream stabilizers to compensate for damage introduced during manufacture [20,34,79].

### 5.2. Use Reversible Retention Rather than Indiscriminate Immobilization

When a protein escapes too rapidly from an otherwise compatible aqueous matrix, a specific reversible interaction can provide transport control without requiring extreme physical confinement. The Fc-binding hyaluronan system developed by Isaacs-Bernal et al. reduced the release and effective diffusivity of Fc-Noggin and anti-sFRP2 while preserving their respective biological activities [84]. Because the therapeutic remained non-covalently associated with the matrix, retention could be introduced without permanently modifying the protein [84]. Coordination-based interactions provide another emerging route for controlling biomacromolecular association with delivery matrices; metal-ion-mediated systems have been developed for proteins and peptides through strategies including biomimetic mineralization, metal–organic frameworks, and metal-controlled hydrogel architectures [103].

Affinity should nevertheless be tuned rather than maximized. A useful formulation must demonstrate that increased retention is accompanied by adequate recovery and preserved cargo-specific function. Accordingly, release kinetics, cumulative recovery, and post-release potency should be considered together when selecting an affinity-mediated long-acting system [84].

### 5.3. Engineer the Microenvironment, Not Only the Bulk Material

The microenvironment experienced by a therapeutic inside a degrading depot can differ substantially from the surrounding physiological environment. Hong et al. mapped the microclimate pH of exenatide-loaded PLGA microspheres after subcutaneous implantation for up to 6 weeks and showed that the in vivo pH trajectory differed markedly from that observed under conventional high microsphere concentration in vitro release conditions [82]. Mass-spectrometric analysis simultaneously demonstrated progressive degradation of retained exenatide, dominated by pH-dependent acylation [82]. These findings directly connect the evolving depot microenvironment with chemical modification of the therapeutic and show why bulk external pH is an inadequate surrogate for the conditions experienced inside the carrier.

Carrier chemistry can also be modified to mitigate a defined degradation pathway. Ghassemi et al. compared octreotide release from conventional PLGA with the more hydrophilic polyester PLHMGA [30]. Although PLGA provided prolonged release, more than 75% of the peptides released from PLGA were acylated adducts, whereas PLHMGA formulations substantially increased the fraction of native octreotide recovered during sustained release [30]. Together, these studies show that microenvironmental engineering should target the experimentally demonstrated cargo liability rather than the bulk material class alone. For acylation-prone peptides, internal pH and reactive polyester chemistry are therefore more informative design variables than release duration in isolation [30,82].

### 5.4. Separate Stabilization from Release Control

A recurring opportunity is hierarchical formulation, in which one component establishes a cargo-compatible state or primary diffusion barrier while a second component controls bulk transport from the depot. Importantly, pre-encapsulation improves long-acting delivery only when the added compartment addresses a specific release or stability limitation; introducing an additional particle phase is not intrinsically beneficial. Zheng and Doyle [80] demonstrated this principle using high-concentration amorphous solid IgG encapsulated in alginate microparticles and subsequently dispersed in a thermogelling methylcellulose matrix. At a formulation concentration of 150 mg/mL, unmodified alginate microparticles alone released essentially 100% of the antibody within 1 h, whereas incorporation of the microparticles into the methylcellulose-containing composite reduced the 1 h release to approximately 1–2% or less. With 6%-modified alginate particles in the thermogelling matrix, the reported time to 80% release was 2.6 days, while the released antibody showed no significant change in monomer percentage relative to the control [80].

The broader applicability of this strategy was demonstrated by Zhang et al. [83], who pre-encapsulated G-CSF, GM-CSF, IGF-1, factor VIII (FVIII), and BSA in dextran microparticles before dispersing them in a PLGA–PEG–PLGA thermogelling depot. For G-CSF, GM-CSF, and BSA, initial release was reduced to ≤5% within 30 min compared with >25% when the proteins were incorporated directly into the thermogel. FVIII showed <1% release during the first day compared with >20% without microparticle pre-encapsulation, while the released proteins retained ≥90% of their native biological activity. In rats, the FVIII particle-containing formulation reduced the peak plasma concentration from 132.63 ± 66.67 to 88.25 ± 30.21 ng/mL and prolonged concentrations above the reported minimum therapeutic level from 15 to more than 18 days. These results show that a particle-within-depot architecture can suppress burst release while preserving protein function and, in an appropriate system, translate the altered release profile into prolonged in vivo exposure.

This benefit, however, should not be generalized to every microencapsulation strategy. A secondary particle phase introduces additional interfaces, manufacturing steps, and potential barriers to complete recovery, and degradable polyester particles can themselves generate stability-relevant microenvironments. The design decision should therefore be cargo-specific: pre-encapsulation is most attractive when the primary particle protects the therapeutic or suppresses rapid escape, while the surrounding matrix provides a second, independently tunable transport barrier. Composite architectures can thereby decouple high loading, cargo stabilization, burst suppression, injectability, and residence time, but their increased manufacturing and analytical complexity must be justified by a demonstrated improvement in therapeutic integrity or exposure.

### 5.5. Exploit Cargo Self-Assembly Only When the Assembled State Is Controlled

Self-assembly can convert a therapeutic peptide into its own long-acting reservoir and reduce inert carrier burden. The cotadutide study combined reversible peptide self-assembly with an in situ forming low-molecular-weight PLGA depot to control burst and prolong delivery [99]. This strategy highlights a broader principle: molecular state can be part of release engineering. It also raises a corresponding integrity requirement. The assembled, stored, and released states must each be characterized, because a self-assembled reservoir that contains irreversible aggregates or off-pathway species would not meet the same pharmaceutical objective.

### 5.6. Engineer for the Host–Depot Interface

Host response can no longer be treated solely as a toxicity screen after release optimization. The sustained-antibody hydrogel work by Jons et al. demonstrates that infiltrating cells can alter transport and that modifying local inflammation can extend exposure [81]. This suggests a new materials objective: host-aware release engineering. The relevant variables may include cell infiltration, matrix remodeling, protein adsorption, local enzymes, fibrosis, and tissue mechanics. These effects are likely to be platform- and site-dependent, so they should be incorporated where direct evidence supports a performance impact rather than generalized to every depot.

The design principle is a compatibility window, not a universal material ranking. Development should follow a sequence. First, identify the cargo’s dominant failure modes. Next, select a retention mechanism that does not intensify them. Engineer the local microenvironment and choose a processing route that preserves the drug. Finally, verify that the intended residence interval remains inside the drug’s demonstrated integrity window.

### 5.7. Selecting a Matrix According to Protein Liability and Product Goal

There is no universally optimal matrix for prolonged protein delivery, but the available evidence supports a practical hierarchy for formulation selection. For a structurally labile protein, particularly an antibody or other large folded protein, an aqueous injectable hydrogel is generally a rational starting environment because it can avoid organic-solvent exposure and the acidic degradation products associated with polyester matrices. A simple hydrogel, however, is often insufficient for highly hydrophilic proteins because mesh diffusion can produce rapid escape. In such cases, the preferred design is an aqueous matrix supplemented with a tunable retention mechanism, such as reversible affinity or a hierarchical particle-within-hydrogel architecture. Affinity-functionalized hyaluronan hydrogels, for example, have provided sustained delivery of antibody-based therapeutics while preserving drug-specific biological activity, and a single affinity-depot administration produced an in vivo response comparable to three repeated bolus administrations [84]. For high-concentration antibody delivery, composite systems in which solid antibody is first incorporated into alginate microparticles and then dispersed in a thermogelling methylcellulose matrix provide another useful architecture because loading, injectability, and release control can be adjusted through different formulation components [80].

Polyester depots remain appropriate when substantially longer residence is required, and the therapeutic can be stabilized against processing and degradation-associated stresses. This distinction is demonstrated by successful protein formulations rather than by polymer class alone. Zinc-complexed recombinant human growth hormone encapsulated in PLGA microspheres maintained protein integrity and produced elevated serum concentrations for up to one month, more than 20-fold longer than the corresponding solution administration [34]. Likewise, spray-dried Fab1 incorporated into an injectable PLGA–triacetin depot exhibited sustained release for nearly 80 days while retaining more than 80% antigen-binding capacity after several weeks [31]. These examples show that PLGA can be compatible with labile proteins when the protein state, manufacturing process, polymer properties, and local microenvironment are deliberately engineered.

Accordingly, matrix selection should begin with the dominant liability of the therapeutic rather than with a preferred material class. Proteins that are highly sensitive to solvent exposure, acidic microenvironments, or covalent modification should initially favor aqueous hydrogel-based systems, with affinity or hierarchical retention added when diffusion is too rapid. High-dose antibodies may particularly benefit from composite systems that separate a protein-stabilizing state from the outer transport-controlling network. In contrast, proteins requiring very long release periods may justify PLGA-based systems when solid-state stabilization and microenvironmental control can preserve the relevant quality attributes. The most appropriate matrix is therefore the one that provides the required exposure duration while keeping the therapeutic within its experimentally demonstrated integrity window.

## 6. Proving Therapeutically Competent Release: Beyond the Cumulative Release Curve

A mechanistically sophisticated material can still be evaluated with an analytically weak endpoint. This is one of the largest gaps in the field. Cumulative release measured by ultraviolet (UV) absorbance, total protein assay, radioactivity, or nonspecific fluorescence answers a mass-balance question; it does not automatically answer whether the released material remains the intended medicine. The analytical package should therefore scale with the therapeutic claim.

### 6.1. Mass Recovery and Release Kinetics (Level 1)

Mass recovery is indispensable because it establishes release completeness, burst, lag phases, and material balance. However, methods must be specific enough to distinguish the drug from degradation products where that distinction matters. A total protein assay is useful for recovery but cannot resolve aggregates or covalent variants. Fluorescent labels can alter transport and may remain detectable after molecular damage. Radiolabels can track location without proving native structure. These methods should be recognized for what they measure rather than treated as surrogates for potency.

### 6.2. Chemical Identity and Purity (Level 2)

For peptides, chromatographic and mass-spectrometric methods are often central because they can reveal oxidation, hydrolysis, deamidation, truncation, or polymer-associated acylation [30,71,72,73,74,75]. Protein therapeutics may require peptide mapping or other stability-indicating methods depending on the known degradation pathways. The key principle is specificity: an assay should resolve the molecular change that the depot is most likely to produce.

### 6.3. Physical and Higher-Order Structural Integrity (Level 3)

Protein release should be interrogated with orthogonal methods appropriate to the molecule. Size-exclusion chromatography can quantify soluble monomer and high-molecular-weight species; light-scattering or particle methods characterize association and particulate burden; circular dichroism, fluorescence, infrared spectroscopy, or other biophysical methods can probe higher-order structure. No single technique establishes native state across all proteins. The analytical panel should be justified by the molecule’s critical quality attributes and the suspected material-induced stress [32,33,38,47,48,49,50].

### 6.4. Target-Specific Biological Function (Level 4)

Recent studies show how this evidence ladder can be applied more rigorously. The 2024 single-domain antibody hydrogel study linked depot release with in vivo imaging and therapeutic localization [78]. Newer antibody hydrogel studies increasingly combine release measurements with structural, functional, or PK readouts [80,81,84]. A particularly informative example is provided by Isaacs-Bernal et al. [84], who used an Fc-binding peptide-functionalized hyaluronan hydrogel to introduce reversible affinity-mediated retention of anti-sFRP2 and Fc-Noggin. Affinity functionalization reduced the cumulative release and effective diffusivity of both therapeutics relative to the non-affinity network, illustrating how molecular recognition can supplement mesh-controlled transport without covalent modification of the therapeutics [84]. Importantly, the study extended evaluation beyond release kinetics. Anti-sFRP2 recovered after seven days retained activity in a retinal stem-cell sphere assay, whereas released Fc-Noggin maintained its ability to suppress BMP4-associated alkaline phosphatase activity. In vivo, a single affinity-hydrogel administration increased the Grm7^+^EdU^+^ retinal stem-cell population to an extent comparable to repeated bolus administration, while a single bolus was insufficient [84]. Repeated injections were also associated with reduced outer nuclear layer thickness, whereas this change was not observed with the single affinity-depot treatment. Together, these observations connect material-mediated retention with cargo-specific biological function and an in vivo response rather than inferring therapeutic performance from cumulative release alone [84].

The evidence boundary is equally important. Although the released therapeutics retained drug-specific biological activity, functional preservation does not establish complete molecular integrity. Protein recovery was assessed largely by immunochemical methods, and the study did not report a comprehensive stability-indicating package resolving chemical variants, monomer/aggregate state, and higher-order structure of the released proteins. Moreover, lower cumulative release from an affinity network cannot by itself distinguish productive reversible retention from incomplete recovery or a slowly exchanging bound population. This study therefore provides strong evidence for functional integrity and meaningful in vivo support toward therapeutic competence, but it does not independently establish every preceding level of the proposed Therapeutic Integrity Evidence Ladder. This distinction reinforces a central principle of this Review: the strength of a sustained-delivery claim should match the molecular and pharmacological evidence used to support it.

### 6.5. The In Vivo Therapeutic Competence (Level 5)

The strongest claim—successful long-acting therapy—requires evidence that the formulation produces the intended exposure and pharmacological effect without unacceptable safety liabilities. This does not mean every materials paper must perform a full efficacy program. It means that wording should match evidence. ‘Sustained release’ is a kinetic observation; ‘sustained delivery of active therapeutic’ is a stronger pharmaceutical claim; ‘long-acting therapy’ additionally implies in vivo exposure and function.

### 6.6. A Proposed Therapeutic Integrity Evidence Ladder

We propose the Therapeutic Integrity Evidence Ladder as a claim–evidence framework rather than as a validated scoring system or universally established hierarchy. It was not derived from a formal meta-analysis of the frequency or completeness of analytical evidence across material classes, and the five levels should not be interpreted as mandatory sequential stages that every long-acting formulation study must satisfy. Instead, the framework distinguishes the type of evidence supporting a particular claim: mass detected, chemical integrity, physical or structural preservation, biological activity, and in vivo pharmacological performance. A study may provide convincing evidence in one of these domains while leaving others incompletely characterized.

The experimental literature illustrates this heterogeneity. Ghassemi et al. directly resolved native and acylated octreotide released from degrading polyester microspheres, providing chemical-integrity evidence that would have been obscured by cumulative mass release alone [30]. Zhang et al. combined sustained-release measurements with protein-specific biological activity assays and in vivo pharmacokinetics, thereby extending the evidence beyond mass recovery for several model proteins [83]. Isaacs-Bernal et al. similarly coupled affinity-controlled antibody release with drug-specific biological assays and an in vivo retinal response, while not providing a comprehensive analytical package resolving every possible chemical and higher-order structural variant of the released proteins [84]. These examples demonstrate why evidence packages should be interpreted according to what was actually measured rather than assumed to progress uniformly through all five levels.

Figure 4 summarizes the proposed Therapeutic Integrity Evidence Ladder and visually distinguishes the five evidence domains from nominal mass release to in vivo pharmacological performance. Table 4 complements this framework by mapping each evidence domain to the corresponding scientific question, representative analytical approaches, limitations of the individual evidence type, and the level of claim that can reasonably be supported. The purpose of the proposed ladder is therefore to expose analytical gaps and align the strength of a long-acting-delivery claim with the evidence used to support it, not to imply that the framework itself has already been validated across all material–cargo combinations.

**Table 4:** Proposed evidence domains for evaluating claims of therapeutically competent release.

Evidence Level	Question Answered	Representative Methods	What It Cannot Prove Alone	Recommended Claim Language
1. Mass/kinetics	How much nominal cargo left the depot, and when?	Drug-specific HPLC/UPLC where possible; total protein; validated label-based tracing	Chemical identity, monomer state, higher-order structure, potency	“Sustained mass release”
2. Chemical integrity	Is the released molecule chemically intact?	LC–MS, peptide mapping, stability-indicating chromatography, variant analysis	Native conformation or biological function	“Sustained release of chemically intact drug”
3. Physical/structural integrity	Is the protein monomeric and appropriately folded?	SEC, DLS/particle analysis, CD, intrinsic fluorescence, FTIR, or orthogonal biophysics	Target binding or cellular potency	“Structurally preserved release”
4. Functional integrity	Does released cargo perform its intended molecular/biological function?	Binding assays, enzymatic assays, receptor assays, cell-based potency	In vivo exposure, duration, clinical relevance	“Sustained release of biologically active cargo”
5. In vivo competence	Does the formulation produce the intended exposure and effect?	PK, biodistribution where relevant, PD, efficacy, safety/immunogenicity assessment	Broad clinical generalizability without appropriate models	“Long-acting therapeutic performance”

The levels represent proposed evidence domains rather than a validated scoring system or mandatory sequential testing scheme. Methods are illustrative rather than compulsory panels and should be selected according to cargo-specific critical quality attributes, anticipated degradation pathways, and the strength of the therapeutic claim.

## 7. From Depot Release to In Vivo Exposure and Pharmacological Effect

In vitro release and in vivo exposure are not interchangeable. A depot can release slowly in vitro, yet produce faster or more variable exposure in vivo. The opposite can also occur when systemic persistence masks changes in depot release. This distinction is especially important for antibodies, for which subcutaneous absorption and bioavailability are governed by tissue transport as well as formulation properties [62,97,104].

### 7.1. In Vitro Release Is Not Automatically In Vivo Release

IVIVC remains difficult for complex long-acting systems. Manufacturing-induced differences in peptide-loaded PLGA microspheres can alter both in vitro and in vivo performance [105]. Burst behavior can also be represented differently in vitro and in vivo [106]. Recent LAI literature continues to emphasize the need for discriminatory, mechanistically relevant release tests and careful interpretation of accelerated methods [13,15,16]. Stability-aware IVIVC should additionally ask whether the same molecular species are being compared across both settings.

Hydrogels present a related but different challenge. Network dilution, mechanical deformation, tissue fluid exchange, enzymatic remodeling, and host–cell infiltration can change release relative to a static buffer study. Earlier work comparing protein release from hydrogel systems in vitro and in vivo already demonstrated the importance of physiological context [107]. The recent antibody depot study takes this further by showing a mechanistic role for cellular infiltration in accelerating transport [81].

### 7.2. Release Rate, Absorption Rate, and Elimination Half-Life Are Separable

For systemic long-acting therapy, the observed concentration–time profile reflects at least three kinetic layers: release from the depot, absorption from the injection site, and elimination of the drug from circulation. If release is much slower than elimination, the depot can become the controlling input. If the therapeutic has a very long systemic half-life, modest changes in depot release may have less influence on the terminal profile. Pharmacokinetic models for long-acting injectables therefore need drug physicochemical and biological properties in addition to material release parameters [108]. Jons et al. explicitly modeled how antibody elimination half-life and hydrogel depot volume influence projected PK [81], illustrating the value of connecting material design to exposure rather than treating them as separate disciplines.

### 7.3. PK Should Track Active Species When Integrity Changes

A deeper issue arises when the depot releases an altered drug. Conventional PK quantification may count parent-like, immunoreactive, or labeled material that is not fully potent. If the stability–release paradox is significant, a model based on total detectable concentration can overestimate pharmacologically relevant exposure. The appropriate solution is molecule-specific: use analytical methods capable of distinguishing intact from modified species, or pair PK with potency-relevant assays. This is particularly important for peptides susceptible to covalent modification and for proteins in which binding competence can be lost without complete disappearance of assay signal.

### 7.4. Local Delivery Requires Spatial Pharmacology

For local depots, plasma concentration may be a poor surrogate altogether. The relevant variable can be the concentration of the active therapeutic in the target tissue as a function of space and time. Affinity hydrogels used for growth-factor delivery illustrate the importance of local retention [94,95]. Antibody fragments released near tumors similarly depend on residence at the target as well as systemic persistence [78]. Future long-acting material studies should therefore define whether the product goal is systemic exposure, local tissue exposure, or both, and select release and PK/PD measurements accordingly. Figure 5 connects therapeutically competent release to the exposure processes that determine PK and PD in vivo.

## 8. Translation: From an Elegant Material to a Viable Medicine

### 8.1. Clinically Meaningful Dose and Loading

Translation begins with dose arithmetic. A depot that achieves exquisite release of microgram quantities may not solve a product problem that requires tens or hundreds of milligrams. Peptide products can be highly potent, but antibodies frequently require high doses and face strict subcutaneous volume constraints [36,37]. The formulation therefore needs a credible path to clinically relevant drug loading without intolerable viscosity, excessive injection force, burst, or cargo destabilization. High-concentration hydrogel systems [80] and long-acting incretin depots [85,99] are important because they address dose and formulation practicality rather than demonstrating release only at analytical-scale concentrations.

### 8.2. Injectability, Administration Route, and User Burden

Injectability is not a secondary device issue. It is part of formulation feasibility. Shear-thinning and in situ-forming hydrogels can reduce administration burden [85,92]. Microsphere suspensions and in situ-forming polymer solutions create different constraints around particle size, resuspension, solvent tolerance, and needle passage [11,12,13,14,15,16,17,18,19]. High-concentration antibodies add viscosity and dose-volume constraints that can dominate product design [59,60,61,62,63,64,65,66,67].

### 8.3. Manufacturing Reproducibility and QbD

Long-acting performance is unusually sensitive to small changes in material and process attributes. For PLGA products, molecular weight distribution, end group, monomer ratio, blockiness, particle size, porosity, and internal drug distribution can all influence release [101,105,106,109,110,111]. QbD provides a disciplined way to connect the quality target product profile to critical quality attributes, material attributes, and process parameters [109]. The same logic should be extended to advanced hydrogels, where polymer substitution, crosslink density, particle content, rheology, gelation kinetics, and affinity-ligand density can become release-critical attributes.

### 8.4. Shelf Stability and In-Depot Stability Are Different Problems

A long-acting formulation must survive two distinct clocks. The packaged product must remain stable through manufacture, shipping, and shelf life; after administration, the therapeutic must remain acceptable throughout depot residence. A formulation can pass conventional shelf stability while failing after weeks in an evolving polymer microenvironment, or it can perform well in vivo but be impractical to store. Carrier preservation can itself become a formulation variable in biologically derived delivery systems; for extracellular vesicles, storage-buffer composition has been shown to influence vesicle preservation and delivery-relevant properties during storage [112]. ICH Q5C provides stability principles for biotechnological/biological products, and Q6B frames specifications and test procedures for relevant recombinant protein and polypeptide products [113,114]. These guidelines do not prescribe how to qualify every novel depot, but they reinforce the need for stability-indicating methods tied to product quality attributes.

### 8.5. Sterility, Aseptic Processing, and Material–Drug Compatibility

Manufacturing compatibility must be shown for the complete material–biologic product. Changes to the matrix can alter drug stability, release, or both. Compatibility, therefore, cannot be inferred from the preprocessed ingredients. Quality-by-Design and risk-management frameworks are useful because they force links among material attributes, process parameters, release behavior, and product quality [13,15,101,109].

### 8.6. Immunogenicity and Safety Require Disciplined Claims

Aggregates, particles, chemical modifications, impurities, and host responses can contribute to immunogenicity risk, but none is a deterministic predictor of clinical immunogenicity. Experimental and review literature supports a cautious, product-specific interpretation of aggregate-related risk [55,56,57,58]. FDA and EMA guidance likewise emphasizes risk-based assessment of product-, patient-, and treatment-related factors. Claims in this area should therefore distinguish a quality concern from a demonstrated clinical immune outcome.

### 8.7. Critical Quality Attributes Should Span Drug, Material, and Combination State

The regulatory challenge is that the therapeutic and material cannot be characterized independently when their interaction determines performance. Drug-related attributes include identity, purity, monomer/aggregate profile, higher-order structure where relevant, and potency. Material attributes include composition, molecular-weight distribution, crosslinking or substitution, degradation, residuals, mechanics or rheology, and sterility. Combination-state attributes include loading, spatial drug distribution, drug–material affinity, microenvironmental conditions, release kinetics, and integrity of the released product. Analytical procedures must be fit for purpose and appropriately validated. Sterility and particulate control add further product-specific requirements [115]. A future regulatory framework for long-acting biologics must connect these layers rather than treating release testing as a stand-alone quality endpoint. Table 5 summarizes translational failure modes that should be addressed before a high-performing depot can be considered a viable pharmaceutical product.

## 9. A Target-Product-Profile-Driven Framework for Long-Acting Peptide and Protein Therapeutics

The field does not need a single ‘best’ depot material. It needs a disciplined way to decide which material architecture is compatible with a specific medicine and therapeutic objective. We propose an integrity-aware target-product-profile framework that starts from clinical exposure and cargo vulnerability rather than from a favored polymer.

### 9.1. Define the Medicine Before Selecting the Material (Step 1)

Map molecular size, charge, hydrophobicity, concentration dependence, known chemical degradation pathways, aggregation propensity, higher-order structural requirements, potency, dose, and acceptable route. For peptides, explicitly assess nucleophilic residues and known polyester-reactive liabilities when degradable aliphatic polyesters are considered [30,71,72,73,74,75]. For proteins and antibodies, define monomer, particle, conformation, and potency attributes relevant to the product [32,33,35,36,37,38,47,48,49,50,55,79].

### 9.2. Define the Exposure Target (Step 2)

Specify the desired duration, acceptable initial burst, minimum and maximum exposure, and local versus systemic target. Also decide whether the depot must be reversible or retrievable. A weekly-to-monthly peptide product and a months-long antibody prophylaxis impose different constraints, even though both are ‘long-acting.’ The exposure target should determine the required release input. It should not be retrofitted after material selection [18,19,63,97,104].

### 9.3. Map the Stability Window Inside Candidate Microenvironments (Step 3)

Measure cargo integrity under material-relevant pH, water activity, ionic strength, concentration, interfaces, degradation products, and temperature over the intended residence period. Static solution stress studies are useful screening tools, but should be complemented by measurements of cargo remaining inside the real material. Spatially resolved microenvironment measurements are particularly valuable for degradable matrices [82,86,87,88,89].

### 9.4. Choose a Retention Mechanism Compatible with the Cargo (Step 4)

Diffusion control, degradation, reversible affinity, self-assembly, or hierarchical compartmentalization should be selected according to the cargo’s stability map. Strong affinity is appropriate only when binding remains reversible and functionally neutral [84,93,94,95,96,98]. Degradation-controlled release is appropriate when the cargo tolerates or is protected from the evolving microenvironment [10,11,12,13,14,62,63,64,65,66,67,76,77,78,79,80]. Hierarchical systems are useful when stabilization and release need to be decoupled [80,85,90,91].

### 9.5. Apply Residence-Time–Stability Matching (Step 5)

The intended residence interval should be compared with the onset of unacceptable integrity loss under the evolving depot conditions. This does not require a single universal ‘damage time.’ Different critical attributes may fail at different rates, and the acceptance boundary is product-specific. The practical output is a compatibility map showing whether the proposed release duration sits inside or outside the cargo’s demonstrated stability window.

### 9.6. Demonstrate Competent Release and Connect It to PK/PD (Step 6)

Use the Therapeutic Integrity Evidence Ladder to decide which analytical levels are necessary for the intended claim, then connect release to exposure with in vivo measurements and mechanistic modeling [105,106,108,110,111]. If the depot changes drug integrity, PK should distinguish active from merely detectable species where technically possible. Local products should quantify target-tissue exposure rather than relying exclusively on plasma concentrations.

### 9.7. Priority Experiments for the Next Phase of the Field (Step 7)

Several experiments would move the field beyond descriptive release studies. First, head-to-head material comparisons should use the same therapeutic, dose, and integrity panel so that material effects are separable from cargo differences. Second, depot microenvironments should be measured spatially and temporally in vivo, extending the type of microclimate mapping demonstrated for PLGA [82]. Third, studies should quantify the integrity of both the released drug and the drug still remaining inside the depot. Fourth, IVIVC should incorporate stability-aware species balance when degradation is significant. Fifth, host–depot interactions should be perturbed experimentally when cells, enzymes, or fibrosis are suspected to control release [81]. Sixth, clinically realistic loading and injection constraints should be used earlier, because dilute proof-of-concept systems can hide concentration-dependent failure. Finally, standardized reporting of material attributes, cargo quality, assay specificity, and release conditions would create datasets suitable for predictive modeling without replacing mechanistic validation.

These priorities also clarify where artificial intelligence and computation can be useful. Predictive models may eventually integrate cargo sequence/structure, material chemistry, microenvironment, and release to prioritize formulations. At present, however, the field lacks sufficiently standardized, high-quality datasets across materials and biologics. Computational tools should therefore be used first to organize experimentally anchored variables and identify candidate compatibility regions, not to generate false precision from heterogeneous release curves. Figure 6 provides a target-product-profile-driven workflow for deciding whether a long-acting material is compatible with a specific peptide or protein medicine.

## 10. Conclusions and Outlook

Long-acting peptide and protein delivery is often described as a problem of slowing diffusion or extending polymer degradation. That description is incomplete. For labile therapeutics, the decisive question is whether a material can control residence and release without changing the properties that make the molecule a medicine. Those properties include chemical identity, physical state, structural organization, and biological function. This Review therefore places the stability–release paradox at the center of depot design.

The strongest direct evidence comes from systems that measure microenvironment and cargo transformation together. PLGA studies show that internal pH can diverge markedly from the surrounding medium [82,86,87,88,89]. They also show that peptide acylation can occur during residence [30,71,72,73,74,75]. Newer long-acting PLGA studies reinforce the importance of formulation architecture, pore structure, manufacturing, and bioactivity [11,12,13,14,15,16,17,18,19,90]. Hydrogel and affinity systems demonstrate the complementary opportunity. Aqueous injectable networks can provide gentler environments, while reversible binding, hierarchical structure, or cargo self-assembly can suppress premature escape [20,25,26,34,36,37,38,39,40,41,51,52,53]. Hydrogels are not intrinsically protein-safe, and polyester depots are not intrinsically incompatible. For structurally labile proteins, aqueous affinity or hierarchical hydrogel systems provide a rational starting point when they can prevent rapid diffusive escape, whereas polyester depots remain appropriate when longer residence is required, and the therapeutic can be stabilized against processing and degradation-associated stresses. Matrix selection should therefore be determined by the cargo-specific failure mode, required exposure duration, and experimentally demonstrated integrity window rather than by material class alone.

Three concepts provide a practical way forward. First, the depot should be treated as a dynamic pharmaceutical microenvironment whose pH, hydration, interfaces, local concentration, degradation products, and host interactions evolve with time. Second, release claims should be graded through a Therapeutic Integrity Evidence Ladder so that mass recovery is not confused with the release of active medicine. Third, residence-time–stability matching should be used to test whether the intended dosing interval lies within a demonstrated integrity window for the cargo under relevant depot conditions.

The next generation of long-acting biologic materials should be optimized against a multi-objective target. The formulation must deliver a clinically meaningful dose with acceptable injectability and controlled exposure. It must also preserve therapeutic integrity and remain manufacturable with predictable in vivo performance. These objectives should not be treated as sequential tasks. Material architecture, drug stability, analytical strategy, PK/PD, and CMC must be designed as one coupled system. The relevant endpoint is not the longest release curve. It is the longest period over which the formulation delivers the right molecular species, at the right exposure, with the required biological function.

## Figures and Tables

**Table 1:** Cargo-dependent integrity liabilities and implications for long-acting material design.

Therapeutic Class	Key Attributes to Preserve	Representative Liabilities During Confinement	Design Implication	Refs.
Peptides	Covalent identity; stereochemical/sequence integrity; soluble state; receptor activity	Oxidation, hydrolysis, deamidation, aggregation/self-association, adsorption, polyester acylation	Select retention chemistry that avoids reactive groups; control local pH and water; confirm intact peptide, not only mass	[3,4,30,71,72,73,74,75]
Proteins/enzymes	Higher-order structure; monomer state; catalytic/binding activity	Unfolding, aggregation, surface adsorption, precipitation, clipping, oxidation	Minimize damaging interfaces and harsh processing; use orthogonal structure + activity assays	[32,33,34,38,47,48,49,50,76,77]
Antibody fragments	Binding conformation; monomer state; rapid diffusion/clearance balance	Aggregation, adsorption, incomplete release, short systemic persistence	Pair affinity or network control with direct binding/potency assessment	[31,78]
Monoclonal antibodies	Multidomain structure; monomer; target binding; high-concentration behavior	Self-association, viscosity, interfacial particle formation, aggregation, limited SC volume	Engineer high-loading state and depot independently; avoid treating total IgG recovery as potency	[35,36,37,79,80,81]

SC, subcutaneous. The table identifies representative liabilities; it is not exhaustive, and no liability should be assumed without drug-specific evidence.

**Table 2:** Representative experimental cases linking carrier design, therapeutic integrity, and long-acting performance.

Therapeutic Cargo	Carrier/Depot Architecture	Key Experimental Outcome	Integrity/Function Evidence	Formulation Lesson	Refs.
Octreotide	PLGA microspheres versus more hydrophilic PLHMGA microspheres	PLGA released ~85% of the loading over 95 days, but only ~25% of the released peptide was native, and ~75% was acylated. PLHMGA formulations retained sustained release while increasing the native fraction to >65–70%, depending on copolymer composition.	HPLC and MALDI-TOF resolved native and acylated species.	A satisfactory total-release profile can conceal extensive chemical failure. For nucleophilic peptides, polyester chemistry and the local degradation environment must be optimized together.	[30]
Exenatide	50:50 PLGA microspheres evaluated in vivo	Microspheres were followed after subcutaneous implantation for up to 6 weeks. The in vivo microclimate-pH trajectory differed markedly from conventional high microsphere concentration in vitro testing, while retained exenatide underwent progressive degradation dominated by pH-dependent acylation.	Mass-spectrometric analysis of drug remaining within the depot.	Release-test conditions must reproduce the relevant depot microenvironment; external physiological pH does not establish the pH experienced by retained peptide.	[82]
Recombinant human growth hormone (rhGH)	Zinc-stabilized solid rhGH in PLGA microspheres prepared by a cryogenic, non-aqueous process	Encapsulated rhGH was analytically unaltered relative to pre-encapsulation protein, and a single microsphere administration maintained elevated serum rhGH for up to one month, >20-fold longer than the same dose given as solution.	Chromatographic methods, SDS-PAGE, peptide mapping, receptor-dependent cell proliferation and in vivo exposure.	PLGA is not intrinsically incompatible with proteins; solid-state stabilization and a protein-compatible manufacturing route can change the outcome substantially.	[34]
Fab1 antibody fragment	Spray-dried Fab1 dispersed in PLGA–triacetin injectable depot	Sustained release continued for nearly 80 days.	>80% antigen-binding capacity was retained after several weeks; degradation during depot residence was comparable to long-term incubation in buffer.	For appropriately stabilized protein solids, a polyester depot can provide very long residence without corresponding loss of functional competence.	[31]
Human IgG	High-concentration solid IgG in alginate microparticles dispersed in thermogelling methylcellulose	At 150 mg/mL formulation concentration, unmodified alginate particles released essentially 100% of IgG within 1 h; hydrophobic alginate modification reduced 1 h release to 44.7% and 17.2%. Incorporation into the methylcellulose composite further prolonged release, with t80 reaching 2.6 days for the 6%-modified system.	SEC showed no significant loss of monomer relative to control; released composite formulations remained >80% monomer.	High loading and release control can be separated between a cargo-containing particle and an outer transport-controlling network instead of forcing one homogeneous matrix to satisfy both requirements.	[80]
G-CSF, GM-CSF, IGF-1, FVIII and BSA	Dextran microparticles incorporated into PLGA–PEG–PLGA thermogel	Microparticle pre-encapsulation reduced initial release to ≤5% within 30 min versus >25% for proteins incorporated directly into the thermogel. For FVIII, Cmax decreased from 132.63 ± 66.67 to 88.25 ± 30.21 ng/mL and therapeutic coverage increased from 15 to >18 days.	Released proteins retained ≥90% of native biological activity.	A hierarchical particle-within-depot design is justified when a hydrated thermogel alone is too permeable and causes burst release.	[83]
Fc-Noggin and anti-sFRP2	Fc-binding-peptide-functionalized hyaluronan affinity hydrogel	Fc-binding ligands with approximately 10^−8^ M affinity reduced premature release. A single intravitreal depot administration produced retinal stem-cell activation over 4 days comparable to three repeated bolus injections.	Drug-specific biological activity was demonstrated after hydrogel residence and release.	Reversible molecular affinity can provide retention without extreme mesh restriction, but function of the released protein must be demonstrated rather than inferred from slower diffusion alone.	[84]
Semaglutide and tirzepatide	Injectable polymer–nanoparticle hydrogel depot	A single administration maintained pharmacologically relevant drug levels for >6 weeks in a type 2 diabetes rat model and produced glucose and weight control comparable to repeated free-drug administration.	In vivo PK/PD provides strong functional evidence, although it does not by itself constitute a complete molecular-integrity panel.	For potent peptides where systemic pharmacology can be measured directly, a hydrogel platform can provide convincing long-acting performance, but PK/PD should not substitute for molecule-specific stability analysis when degradation is plausible.	[85]

**Table 5:** Translational bottlenecks and integrity-aware development responses.

Translation Domain	Failure Mode	Why It Matters for Long-Acting Biologics	Integrity-Aware Development Response	Evidence/Guidance
Dose & loading	Release shown only at low, nonclinical loading	May not meet the dose without a large volume or high concentration	Set target dose/volume first; test concentration-dependent stability and release	[36,37,80,85]
Injectability	Excess force, needle blockage, uncontrolled in situ gelation	Can defeat adherence advantage and alter the delivered dose	Measure rheology, injection force, gelation/solidification, and dose recovery	[80,85,92]
Manufacturing	Small material/process changes shift porosity or release	Long-acting release is highly structure-sensitive	Use QbD linking material/process attributes to release and integrity	[101,109]
Shelf vs. in-depot stability	The product is stable in the package, but degrades after injection	The depot creates a different chemical/physical environment	Run time-resolved residual-depot and released-drug stability studies	[30,31,34,71,72,73,74,75,76,77,82,86,87,88,89,114]
IVIVC/PK	In vitro release does not predict absorption/exposure	Host response and absorption kinetics can dominate	Develop mechanism-aware IVIVC/PK models using active species where needed	[81,105,106,108,110,111]
Safety/immunogenicity	Modified or aggregated species and persistent local material	Long exposure may magnify the consequences of quality defects	Risk-based aggregate/particle/ADA strategy; qualified potency and stability assays	[55,116]

QbD, Quality by Design; IVIVC, in vitro–in vivo correlation; PK, pharmacokinetics; ADA, anti-drug antibody. Regulatory requirements are product- and jurisdiction-specific.

## Data Availability

Data are contained within the article.

## Abbreviations

The following abbreviations are used in this manuscript:

ADA	Anti-drug antibody
ALP	Alkaline phosphatase
anti-sFRP2	Antibody against secreted frizzled-related protein 2
BMP4	Bone morphogenetic protein 4
CD	Circular dichroism
CMC	Chemistry, Manufacturing, and Controls
DLS	Dynamic light scattering
EdU	5-Ethynyl-2′-deoxyuridine
EMA	European Medicines Agency
Fc	Fragment crystallizable region
FcL1	Fc-binding peptide ligand 1
FDA	Food and Drug Administration
FTIR	Fourier-transform infrared spectroscopy
Grm7	Glutamate metabotropic receptor 7
HAT-ox	Oxime-crosslinked hyaluronan-based hydrogel
HAT-ox-FcL1	HAT-ox hydrogel functionalized with Fc-binding peptide ligand 1
HPLC	High-performance liquid chromatography
IgG	Immunoglobulin G
IVIVC	In vitro–in vivo correlation
LAI	Long-acting injectable
LC–MS	Liquid chromatography–mass spectrometry
ONL	Outer nuclear layer
PD	Pharmacodynamics
PK	Pharmacokinetics
PK/PD	Pharmacokinetics/pharmacodynamics
PLGA	Poly(lactic-co-glycolic acid)
QbD	Quality by Design
SC	Subcutaneous
SEC	Size-exclusion chromatography
UPLC	Ultra-performance liquid chromatography
UV	Ultraviolet
